# Spiroergometric measurements under increased inspiratory oxygen concentration (FIO2)—Putting the Haldane transformation to the test

**DOI:** 10.1371/journal.pone.0207648

**Published:** 2018-12-12

**Authors:** Stephan Lang, Robert Herold, Alexander Kraft, Volker Harth, Alexandra M. Preisser

**Affiliations:** Institute for Occupational and Maritime Medicine (ZfAM), University Medical Center Hamburg-Eppendorf, Hamburg, Germany; University of Notre Dame Australia, AUSTRALIA

## Abstract

Spiroergometric measurements of persons who require oxygen insufflation due to illness can be performed under conditions of increased inspiratory oxygen concentration (FIO_2_). This increase in FIO_2_, however, often leads to errors in the calculation of oxygen consumption (${\dot{\text{V}}\text{O}}_{\text{2}}$). These inconsistencies are due to the application of the Haldane Transformation (HT), an otherwise indispensable correction factor in the calculation of ${\dot{\text{V}}\text{O}}_{\text{2}}$ that becomes inaccurate at higher FIO_2_ concentrations. A possible solution to this problem could be the use of the ‘Eschenbacher transformation’ (ET) as an alternative correction factor. This study examines the concentration of FIO_2_ at which the HT and the ET are valid, providing plausible data of oxygen consumption corresponding to the wattage achieved during cycle ergometry. Ten healthy volunteers underwent spiroergometric testing under standard conditions (FIO_2_ = 20.9%), as well as at FIO_2_ = 40% and 80%. When compared with the predicted values of ${\dot{\text{V}}\text{O}}_{\text{2}}$, as calculated according to Wasserman et al. (2012), the data obtained show that both the HT and ET are valid under normal conditions and at an increased FIO_2_ of 40%. At FIO_2_ concentrations of 80%, however, the ${\dot{\text{V}}\text{O}}_{\text{2}}$ values provided by the HT begin to lose plausibility, whereas the ET continues to provide credible results. We conclude that the use of the ET in place of the HT in spiroergometric measurements with increased FIO_2_ allows a reliable evaluation of stress tests in patients requiring high doses of supplemental oxygen.

## Introduction

For patients who are dependent on a continuous supply of supplementary oxygen, it is potentially dangerous to perform classical spiroergometry without oxygen insufflation. In order to obtain usable data, testing under conditions of increased inspiratory oxygen concentration (FIO_2_) is necessary. With this method, patients inhale oxygen-enriched air from a gas reservoir. The performance of the patient can thus be determined by means of spiroergometry, without causing unnecessary physical and psychological stress due to breathing difficulties [1, 2]. However, due to the inherent difficulties of such a testing method, the question of practicability remains, namely: "Why do spiroergometric measurements with increased FIO_2_ lead to errors in the calculation of the oxygen uptake (${\dot{\text{V}}\text{O}}_{\text{2}}$)?" [3].

There is consensus in the literature that spiroergometry performed under conditions of increased FIO_2_ leads to an overestimation of the measured ${\dot{\text{V}}\text{O}}_{\text{2}}$. Wasserman summarizes this particular methodological issue in "Principles of Exercise Testing and Interpretation" (2012) [3]. At an FIO_2_ of 40% or greater, the error rate in the calculation of ${\dot{\text{V}}\text{O}}_{\text{2}}$ and the respiratory exchange rate (RER) increases until, finally, under a maximum oxygen concentration of FIO_2_ = 100%, no valid results can be obtained. Similar findings on the inaccuracy of this measurement method are also provided in studies done by Wilmore et al. (1973) [4] and Takala (1991) [5]. A valid ${\dot{\text{V}}\text{O}}_{\text{2}}$ value (i.e., corresponding to actual physical performance) can be assumed if found to be within the tolerated range of ± 10% from the predicted ${\dot{\text{V}}\text{O}}_{\text{2}}$, relative to the achieved physical load (in watts, as calculated according to Wasserman, see ‘Methodology’). In their studies, Prieur et al. [6] and Stanek et al. [7] described this problem of increasing deviation of the measured ${\dot{\text{V}}\text{O}}_{\text{2}}$ from the calculated; at an FIO_2_ ≥ 40%, ${\dot{\text{V}}\text{O}}_{\text{2}}$ measurements were shown to overestimate the level of actual physical performance. ${\dot{\text{V}}\text{O}}_{\text{2}}$ has a linear relationship to the patients load-capacity, e.g. in watts. It is thus possible to deduce the performance of the subject via the ${\dot{\text{V}}\text{O}}_{\text{2}}$ [3, 8]. This held true for varying FIO_2_ concentrations, with an ever-increasing ${\dot{\text{V}}\text{O}}_{\text{2}}$ estimation for increasing FIO_2_ values. The authors also showed a parallel continuous decrease in RER, eventually reaching zero at high FIO_2_ concentrations. Walsh and Banister (1995) [9] performed spiroergometry on seven subjects at varying FIO_2_ concentrations. They found an increased ${\dot{\text{V}}\text{O}}_{\text{2}}$ in relation to load in a stepwise increasing workload protocol under an FIO_2_ of 40%. However, higher FIO_2_ concentrations were not tested in this study. Yet further investigations confirm the effects of increased FIO_2_ on ${\dot{\text{V}}\text{O}}_{\text{2}}$ and RER, but none provide a practical or plausible solution to the problem at higher FIO_2_ concentrations [10–12].

Ulrich et al. [13] explain the disproportionate increase of ${\dot{\text{V}}\text{O}}_{\text{2}}$ to the reason that carbon dioxide emission (${\dot{\text{V}}\text{CO}}_{\text{2}}$) measurements are not influenced by increased levels of FIO_2_, so the RER (presented as a ratio of ${\dot{\text{V}}\text{CO}}_{\text{2}}$ to ${\dot{\text{V}}\text{O}}_{\text{2}}$) is consistently reported as being too low. A possible cause for this problem could be the Haldane transformation (HT), which is used in spiroergometric measurements to calculate oxygen uptake [14]. On the assumption that no nitrogen exchange (VN_2_) takes place, John Scott Haldane (1860–1936) defined a correction factor for the exact calculation of the inspiratory volume (V_I_). V_I_ is elementary for the calculation of ${\dot{\text{V}}\text{O}}_{\text{2}}$, as is the measured expiratory volume (V_E_) [15, 16]. For measurements under normal atmospheric conditions (FIO_2_ = 20.9%), plausible ${\dot{\text{V}}\text{O}}_{\text{2}}$ values (i.e., those corresponding to actual physical performance) can be obtained. This in turn allows for the accurate calculation of the RER. Gunter Kleinberger (1986) [17] showed the limits and weaknesses of ${\dot{\text{V}}\text{O}}_{\text{2}}$ calculations using the HT by demonstrating that various increases of FIO_2_ levels lead to different, nonlinear results: (1) The measurement of ${\dot{\text{V}}\text{O}}_{\text{2}}$ is valid up to an FIO_2_ of 40%. (2) At an FIO_2_ between 40–60%, O_2_ concentration measurements must be repeatedly calibrated. (3) At an FIO_2_ between 60–80%, inaccurate values are to be expected. (4) At an FIO_2_ greater than 80%, implausible data are provided. (5) At an FIO_2_ of 100%, calculation of the ${\dot{\text{V}}\text{O}}_{\text{2}}$ is not possible. As a result of this limitation of the HT, John Hoppe in “Errors in ${\dot{\text{V}}\text{O}}_{\text{2}}$ Testing” (2009) [18], advocates caution when performing spiroergometric tests at FIO_2_ levels greater than 40%.

To achieve a greater level of accuracy regardless of the selected FIO_2_, Hermann Eschenbacher developed the so-called "Eschenbacher Transformation" (ET) in 1987 [19]. When considering these initial series of measurements [14], it seems to allow for plausible calculation of ${\dot{\text{V}}\text{O}}_{\text{2}}$ and RER values at higher FIO_2_ levels.

Following the description of the two computational transformations in terms of their basic theories and implementations [14], the present pilot study will examine the results of both the HT and ET under conditions of increased FIO_2_. The following hypotheses will be discussed:

1. Both transformations yield ${\dot{\text{V}}\text{O}}_{\text{2}}$ and RER values corresponding to the level of actual physical load under normal ambient air conditions (FIO_2_ = 20.9%) and FIO_2_ = 40%.
2. At higher concentrations (FIO_2_ > 40%), the HT produces invalid results in the calculation of ${\dot{\text{V}}\text{O}}_{\text{2}}$ and RER.
3. The ET provides accurate ${\dot{\text{V}}\text{O}}_{\text{2}}$ and RER values even at higher FIO_2_ levels, corresponding to the actual physical load.

## Methods

To test these hypotheses, 10 healthy, middle-aged men underwent spiroergometric examination with three different FIO_2_ concentrations. To participate in the study, a thorough medical assessment including spirometry and diffusion capacity testing was required. Initial stress analyses using classical spiroergometry [20] were carried out under ambient air conditions (FIO_2_ = 20.9%). Subsequent measurements were performed under conditions of increased oxygen concentration (FIO_2_ = 40% and FIO_2_ = 80%), each preceded by a 30-minute rest period. All stress tests were carried out with a stepwise increasing workload protocol on a cycle ergometer (ergoselect 200P, 2016 ergoline GmbH, Bitz, Germany) and evaluated using the HT. After a two-minute reference phase at a load of 20 watts, the applied step protocol provides a ten-minute test phase with five two-minute stages at increasing loads (50-80-110-140-170 watts). The examination terminated with a two-minute recovery phase (20 watts). The so obtained raw data were also used to calculate the ${\dot{\text{V}}\text{O}}_{\text{2}}$ with the ET. The determined values of the ${\dot{\text{V}}\text{O}}_{\text{2}}$ and RER, according to the HT and ET, were then compared. Each stage was evaluated as soon as the steady-state was achieved, usually at the end of the phase. The used HT and ET for ${\dot{\text{V}}\text{O}}_{\text{2}}$ are described with the following calculations:

HT:VO2=VE*kH*FIO2−VE*FEO2

$$kH=\;\frac{\left(1-FEO2-FECO2\right)}{\left(1-FIO2-FICO2\right)}$$

$$ET:\;VO2=\;\frac{\left(VE-VE*\left(FECO2-FICO2\right)\right)*\left(FIO2-FEO2\right)}{1-\left(FIO2-FEO2\right)}$$

The new approach of the ET is based on the complete spectrum of all oxygen concentrations. For this, the ET has to meet four basic conditions: (1) the formula is not based on the assumption that VN_2_ = 0; (2) it takes into account that for RER fraction (which is not equal to one) V_I_ is different to V_E_; (3) the transformation must calculate the values corresponding to the load even with increased FIO_2_; and (4) it must be possible to calculate ${\dot{\text{V}}\text{O}}_{\text{2}}$ at FIO_2_ = 100%. A transformation that fulfills these conditions is a further development of the Haldane Transformation, at least for measurements under elevated FIO_2_. So far there are no verified and validated studies with this transformation.

The prediction values for ${\dot{\text{V}}\text{O}}_{\text{2}}$ according to Wasserman were used as a basis for assessment, with a deviation of ± 10% being acceptable [3, 14]. Wasserman’s plausibility check is the gold standard in the review of ${\dot{\text{V}}\text{O}}_{\text{2}}$. It is a perfect and safe possibility to prove the accuracy of ${\dot{\text{V}}\text{O}}_{\text{2}}$ and it is suited to compare two different transformations affecting ${\dot{\text{V}}\text{O}}_{\text{2}}$, especially at different concentration levels. The predicted ${\dot{\text{V}}\text{O}}_{\text{2}}$ was calculated as follows, incorporating body weight and wattage on the cycle ergometer:

VO2pred.=5.8*BW[kg]+151mL+10.3*load[watts]

From the obtained data, a first view at a potential influence of higher FIO_2_ on the ventilatory threshold was made to investigate further findings.

Spirometry and diffusing capacity studies were performed with the MasterScreen Body/Diffusion and software JLab 5.72, the spiroergometric examinations with the Vyntus CPX and software SentrySuite 2.17, all provided by CareFusion Germany 234 GmbH (2016 CareFusion Germany 234 GmbH, Höchberg, Germany). Masks and equipment were supplied by HansRudolph, Inc. (2014 Hans Rudolph, Inc., Kansas City, MO, USA). Statistical evaluation of the data sets (Mean ± SD, Mean ± SE*2, 2-tailed one sample and paired t-test) was done using the statistics software R (2016 R-Core Team, Vienna, Austria). Images were created using Microsoft Excel (2016, Microsoft Corporation, Redmond, USA). Differences were considered significant at p ≤ 0.05. To correct for multiple comparisons, p-values were adjusted with the Benjamini-Hochberg procedure. Using the Bland-Altman limit analysis [21], the calculated ${\dot{\text{V}}\text{O}}_{\text{2}}$-values for HT and ET were compared with the plausibility formula of Wasserman [3] for predicted ${\dot{\text{V}}\text{O}}_{\text{2}}$ and with it, the proportional bias (p<0.05) was determined by regressing mean differences.

All subjects participated voluntarily and received a small compensation. The participants provide their written informed consent to participate in this study. The Declaration of Helsinki was adequately addressed, and the study was approved by the ethics committee of the Hamburg Medical Association (register number PV5327).

### Spiroergometry with increased FIO_2_

The performance of a spiroergometry with increased FIO_2_ differs from classical spiroergometry only in that the subject breathes a gas mixture with a higher proportion of oxygen compared to indoor climate, from a reservoir equipped with a special valve system. The spiroergometry unit includes ECG leads, blood pressure and pulse oximetry, as well as a respiratory mask. The mask is directly connected to the Digital Volume Transducer and the collection hose for gas measurement. A two-way valve for inhalation and exhalation is connected and is coupled via an adapter to the gas bag into which the subject breathes. The O_2_ filling hose is joined directly to the O_2_ source and the gas bag is filled with the predetermined FIO_2_ concentration through a valve (see also S1 Fig Equipment used). The filling of the gas bag is kept constant during the entire measurement period. At the start of the measurement, the increased FIO_2_ leads to an inflow effect of oxygen into the lung, whereby a disproportionately high ${\dot{\text{V}}\text{O}}_{\text{2}}$ can be observed [14]. The rest phase must therefore be extended until the ${\dot{\text{V}}\text{O}}_{\text{2}}$ value has normalized. The further course follows that of classical spiroergometry [20].

## Results

### Patient group

Ten healthy adult men (n = 10, age: 30 ± 3 years, weight: 80 ± 6 kg, BMI: 24.3 ± 1.3) were included in the study. The medical examination, spirometry, diffusing capacity measurements and the three spiroergometric measurements were carried out from September to December 2016, always over the course of a day. The ambient conditions could be kept almost constant despite the different measuring times (Temperature: 23 ± 2°C, Humidity: 46 ± 6%, Pressure: 1019 ± 2 hPa). All participants fulfilled the inclusion criteria. Table 1 shows the age, body weight, BMI and spirometry values with reference values according to Quanjer et al. (2012) [22]. Table 2 shows the average predicted ${\dot{\text{V}}\text{O}}_{\text{2}}$ value according to Wasserman et al. (2012) [3] for each respective exercise test level.

**Table 1:** Study participants: Mean and range of age, weight and spirometry values in ten healthy men.

Variables*	Mean	Range	%predicted (mean)**
Age, yr	30	27–36	
Weight, kg	80	72–90	
BMI	24.3	22.4–26.6	
TLC, L	7.78	6.50–9.49	105
IC, L	4.22	3.00–5.88	107
FVC, L	6.11	5.55–6.83	107
FEV_1_, L	4.76	4.08–5.40	102

*Abbreviations: *BMI* Body-Mass-Index, *TLC* Total Lung Capacity, *IC* Inspiratory Capacity, *FVC* Forced Vital Capacity, *FEV*_*1*_ Forced Expiratory Volume in one second.

**Reference values according to Quanjer et al. (2012)

**Table 2:** Predicted ${\dot{\text{V}}\text{O}}_{\text{2}}$ for an 80 kg man.

Load [W]	${\dot{\text{V}}\text{O}}_{\text{2}}$ predicted [mL/min]*	Permitted Deviation -10% [mL/min]	Permitted Deviation +10% [mL/min]
50	1128	1015	1241
80	1437	1293	1581
110	1746	1571	1921
140	2055	1850	2261
170	2364	2128	2600

*According to Wasserman et al. (2012)

#### Oxygen uptake and RER

The mean FIO_2_ under standard conditions was 20.78 ± 0.06%. A mean of 39.85 ± 0.18% and 78.57 ± 0.56% FIO_2_ were obtained for the measurements with increased FIO_2_ (40% and 80%, respectively). Table 3 provides an overview of the measured results for ${\dot{\text{V}}\text{O}}_{\text{2}}$ and RER for the three FIO_2_ concentrations.

**Table 3:** Mean (SD) ${\dot{\text{V}}\text{O}}_{\text{2}}$, %Deviation from the Wasserman formula, t-test (${\dot{\text{V}}\text{O}}_{\text{2}}$) and Mean (SD) RER in ten healthy men. Comparison of HT and ET.

FIO_2_ [%]	Load [W]	Haldane Transformation				Eschenbacher Transformation				HT vs ET
		Mean ±SD ${\dot{\text{V}}\text{O}}_{\text{2}}$ [mL/min]	Dev. Was* [%]	p-value (VO_2_)**	Mean ±SD RER	Mean ±SD ${\dot{\text{V}}\text{O}}_{\text{2}}$ [mL/min]	Dev. Was* [%]	p-value (VO_2_)**	Mean ±SD RER	p-value (VO_2_)**
20.78	50	1086 ±84	3.87	0.211	0.81 ±0.03	1024 ±77	10.16	0.014	0.77 ±0.03	0.172
	80	1373 ±72	4.66	0.057	0.86 ±0.06	1314 ±53	9.36	0.001	0.82 ±0.05	0.105
	110	1646 ±58	6.08	0.004	0.92 ±0.08	1584 ±60	10.23	0.000	0.88 ±0.08	0.070
	140	1990 ±96	3.27	0.108	0.99 ±0.07	1898 ±46	8.27	0.000	0.94 ±0.08	0.057
	170	2264 ±112	4.42	0.057	1.01 ±0.08	2222 ±112	6.39	0.017	0.99 ±0.09	0.472
39.85	50	1131 ±121	0.27	0.993	0.70 ±0.13	1030 ±58	9.51	0.004	0.76 ±0.10	0.073
	80	1436 ±200	0.07	0.993	0.75 ±0.14	1319 ±84	8.95	0.012	0.80 ±0.11	0.174
	110	1796 ±298	2.78	0.671	0.80 ±0.17	1677 ±129	4.11	0.187	0.84 ±0.14	0.356
	140	2135 ±329	3.75	0.522	0.83 ±0.17	1987 ±142	3.42	0.233	0.88 ±0.13	0.297
	170	2472 ±319	4.37	0.402	0.90 ±0.17	2375 ±168	0.46	0.903	0.92 ±0.13	0.472
78.57	50	1818 ±718	37.95	0.057	0.45 ±0.20	1016 ±165	11.02	0.108	0.71 ±0.14	0.032
	80	2555 ±1309	43.76	0.062	0.49 ±0.24	1354 ±146	6.13	0.172	0.74 ±0.14	0.057
	110	2891 ±1417	39.61	0.070	0.55 ±0.25	1684 ±207	3.68	0.448	0.78 ±0.15	0.062
	140	3549 ±1644	42.10	0.057	0.55 ±0.26	2054 ±215	0.05	0.993	0.79 ±0.16	0.057
	170	4382 ±2584	46.05	0.073	0.58 ±0.27	2478 ±348	4.60	0.408	0.82 ±0.17	0.089

* %Deviation to the formula of Wasserman et al. (2012) for predicted ${\dot{\text{V}}\text{O}}_{\text{2}}$

** Adjusted for multiple comparisons with the Benjamini-Hochberg procedure

**Fig 1:**
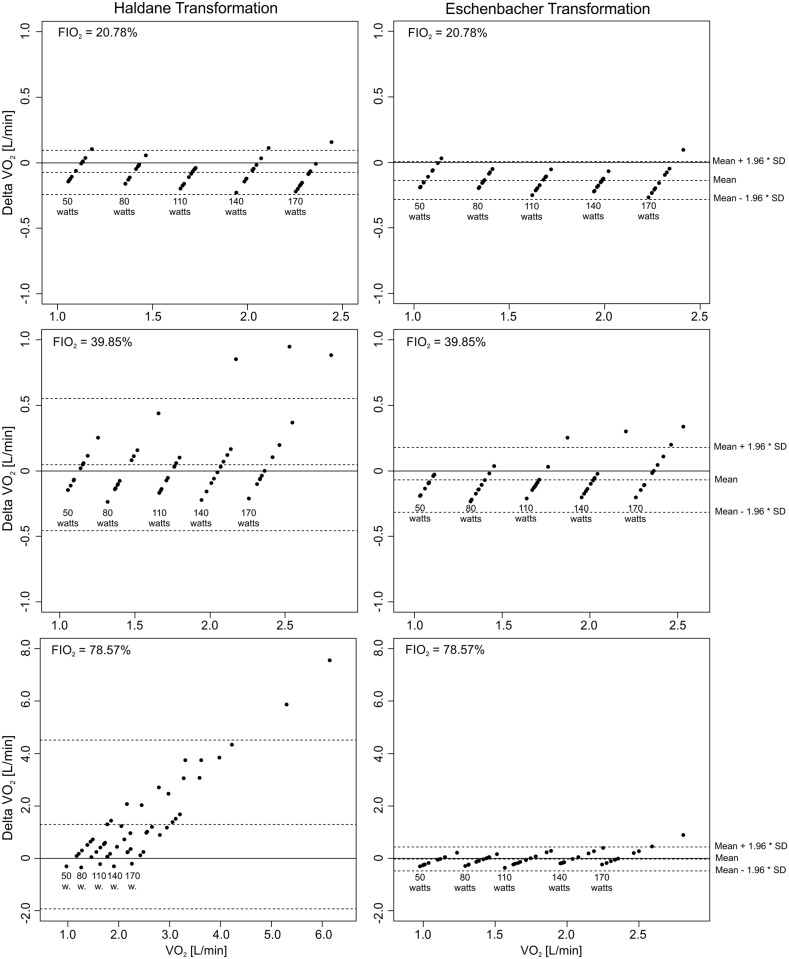
Bland-Altman comparison between HT and ET with the predicted values of Wasserman at each FIO_2_ = Level. On the ordinate, the differences between HT vs. Wasserman and ET vs. Wasserman values are shown. On the abscissa, the reached ${\dot{\text{V}}\text{O}}_{\text{2}}$-values of each participant on each load-level are shown.

Table 4 and Fig 1 provide the Bland-Altman analysis [21] for the comparison of HT and ET with the predicted values of Wasserman et al. (2012) [3] for each FIO_2_-level. At FIO_2_ of 20.78% a similar behavior of both examined transformations is shown. At FIO_2_ = 39.85% and especially at 78.57%, the HT has a much higher fluctuation range than the ET. The ET values show significantly less deviation, but are generally a little lower than the HT values. With increasing FIO_2_-concentration, the ET shows more accurate results (20.78%: p<0.01; 39.85%: p = 0.01; 78.57%: p = 0.87), while the HT shows a constant large dispersion and deviation.

**Table 4:** Bland-Altman limit analysis for comparison between HT and ET for ${\dot{\text{V}}\text{O}}_{\text{2}}$.

Comparison	FIO_2_-Level [%]	Bias ±SD [mL/min]	95% CI [mL/min]	Limits of agreement [mL/min]	Proportional bias (p<0.05)
HT vs.	20.78	-74 ±86	-99 –-50	-243–94	<0.01
Wasserman*	39.85	48 ±258	-26–121	-458–534	0.04
	78.57	1293 ±1644	826–1760	-1928–4514	<0.01
ET vs.	20.78	-138 ±74	-159 –-117	-282–7	<0.01
Wasserman*	39.85	-68 ±126	-104 –-33	-315–178	0.01
	78.57	-29 ±233	-95–37	-485–427	0.87

* HT and ET compared to the predicted values of Wasserman et al. (2012) for ${\dot{\text{V}}\text{O}}_{\text{2}}$

#### FIO_2_ = 20.78%

**Fig 2:**
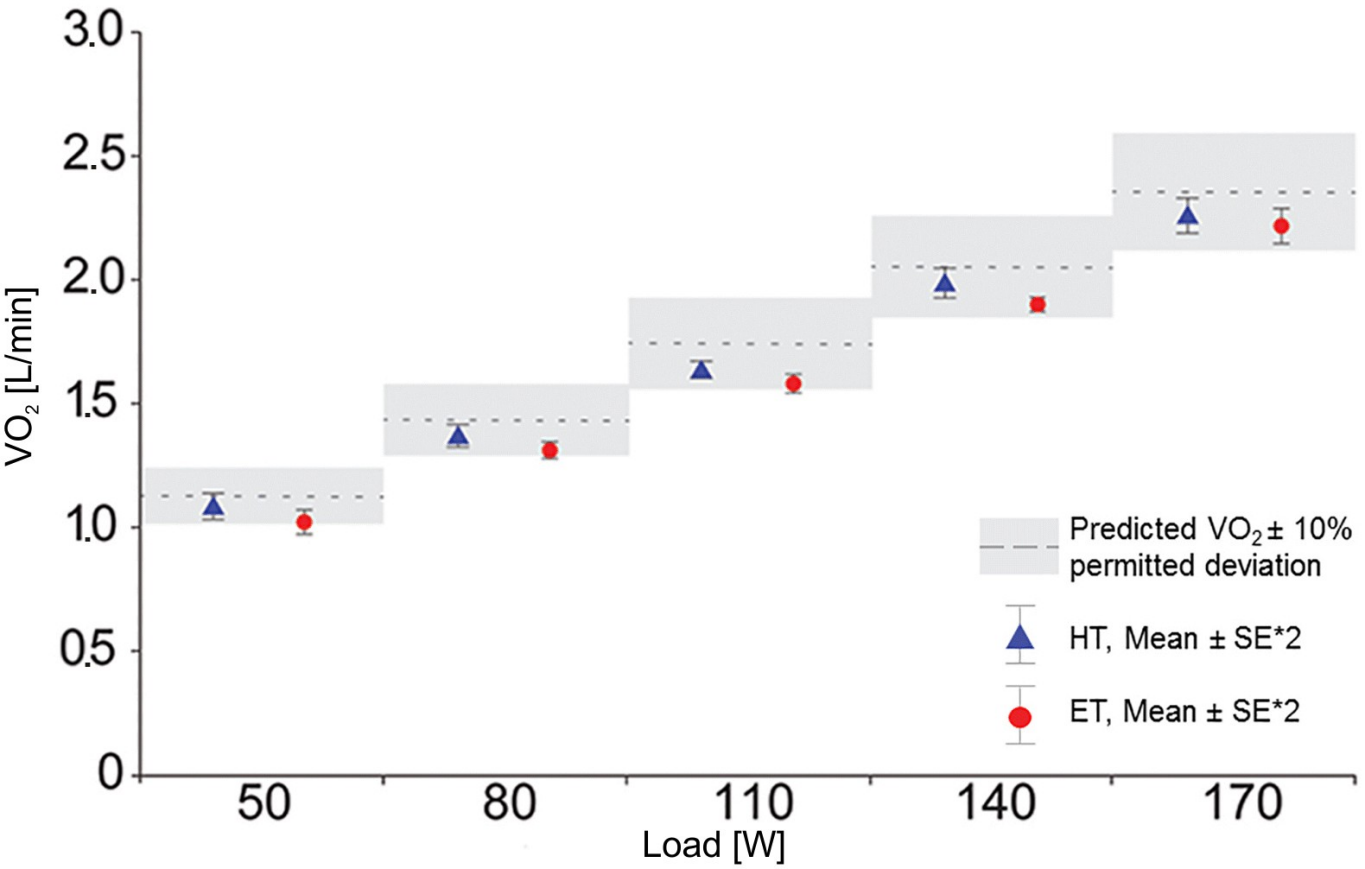
Comparison between HT and ET at FIO_2_ = 20.78%. The dotted lines represent the ${\dot{\text{V}}\text{O}}_{\text{2}}$ predicted for an 80kg man according to Wasserman et al. (2012). The grey block around the dotted line represent the permitted Deviation of ± 10% (see Table 2). The mean values of HT and ET at each load-level are shown with the double standard error to confidence of the plausibility of ${\dot{\text{V}}\text{O}}_{\text{2}}$ predicted. Abbreviations: *HT* Haldane Transformation, *ET* Eschenbacher Transformation.

Under normal ambient air conditions (FIO_2_ = 20.78%), there were only slight differences in the results calculated with the HT and ET. Both transformations provide plausible results for ${\dot{\text{V}}\text{O}}_{\text{2}}$ (Fig 2) and RER. With an average percent deviation of 4.5 ± 1.1% from the predicted ${\dot{\text{V}}\text{O}}_{\text{2}}$ values according to Wasserman (compared to 8.9 ± 1.6% with the ET), the HT appeared to be more accurate. Indeed, overall, Haldane’s results were closer to the calculated values (Table 3). Partially the values of both transformations differed significantly from Wasserman`s predicted values for the specific load levels, with the ET showing a clear deviation below the predicted values. Considering the scattering of the ${\dot{\text{V}}\text{O}}_{\text{2}}$ values, consistent results could be produced with use of the HT as well as the ET (Fig 2; double standard error = 95% confidence of the plausibility of ${\dot{\text{V}}\text{O}}_{\text{2}}$ pred.). Furthermore, the RER values were physiologically plausible in each case.

#### FIO_2_ = 39.85%

**Fig 3:**
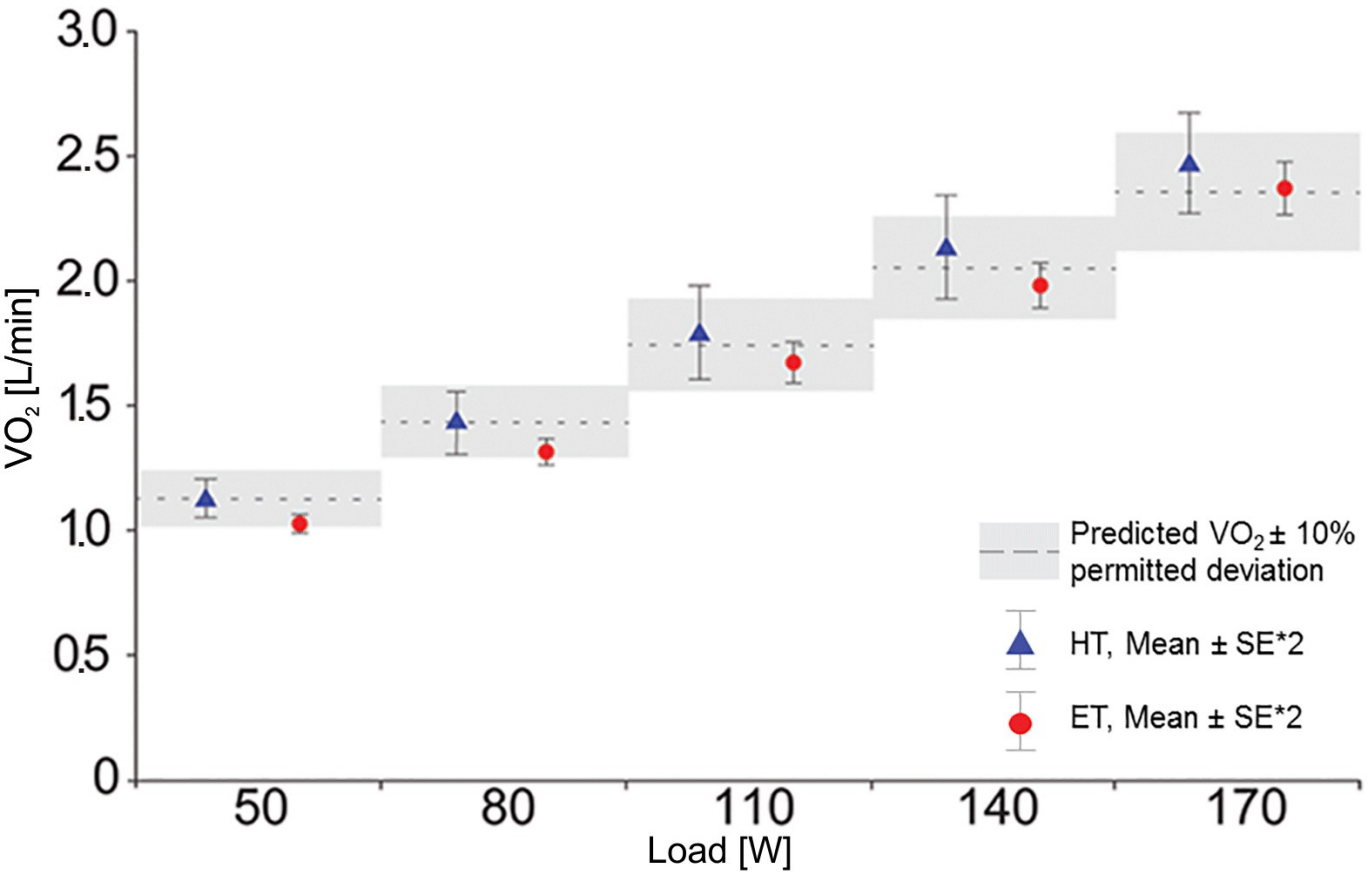
Comparison between HT and ET at FIO_2_ = 39.85%. The dotted lines represent the ${\dot{\text{V}}\text{O}}_{\text{2}}$ predicted for an 80kg man according to Wasserman et al. (2012). The grey block around the dotted line represents the permitted Deviation of ± 10% (see Table 2). The mean values of HT and ET at each load-level are shown with the double standard error to confidence of the plausibility of ${\dot{\text{V}}\text{O}}_{\text{2}}$ predicted. Abbreviations: *HT* Haldane Transformation, *ET* Eschenbacher Transformation.

When FIO_2_ was increased to 39.85%, the measured ${\dot{\text{V}}\text{O}}_{\text{2}}$ values corresponded to the predicted values. The minor deviations of 2.2 ± 2.0% (HT) and 5.3 ± 4.1% (ET) from the predicted ${\dot{\text{V}}\text{O}}_{\text{2}}$ confirmed the applicability of both transformations (Fig 3). Indeed, with respect to predicted ${\dot{\text{V}}\text{O}}_{\text{2}}$ (according to Wasserman), HT and ET values at this level of FIO_2_ were even more consistent on average than at FIO_2_ = 20.78%. Furthermore, the ET produced significantly lower values at 50 and 80 watts than those calculated according to Wasserman (Fig 1). Overall, the results for each transformation showed opposing trends: the HT produced less accurate values with increasing load (> 50 watts), whereas the results of the ET became more accurate (Table 3). The variance of both results, however, increased significantly with load > 50 watts, wherein the HT-calculated values had a greater scattering. At this FIO_2_ concentration, the RER remained physiologically plausible.

#### FIO_2_ = 78.57%

**Fig 4:**
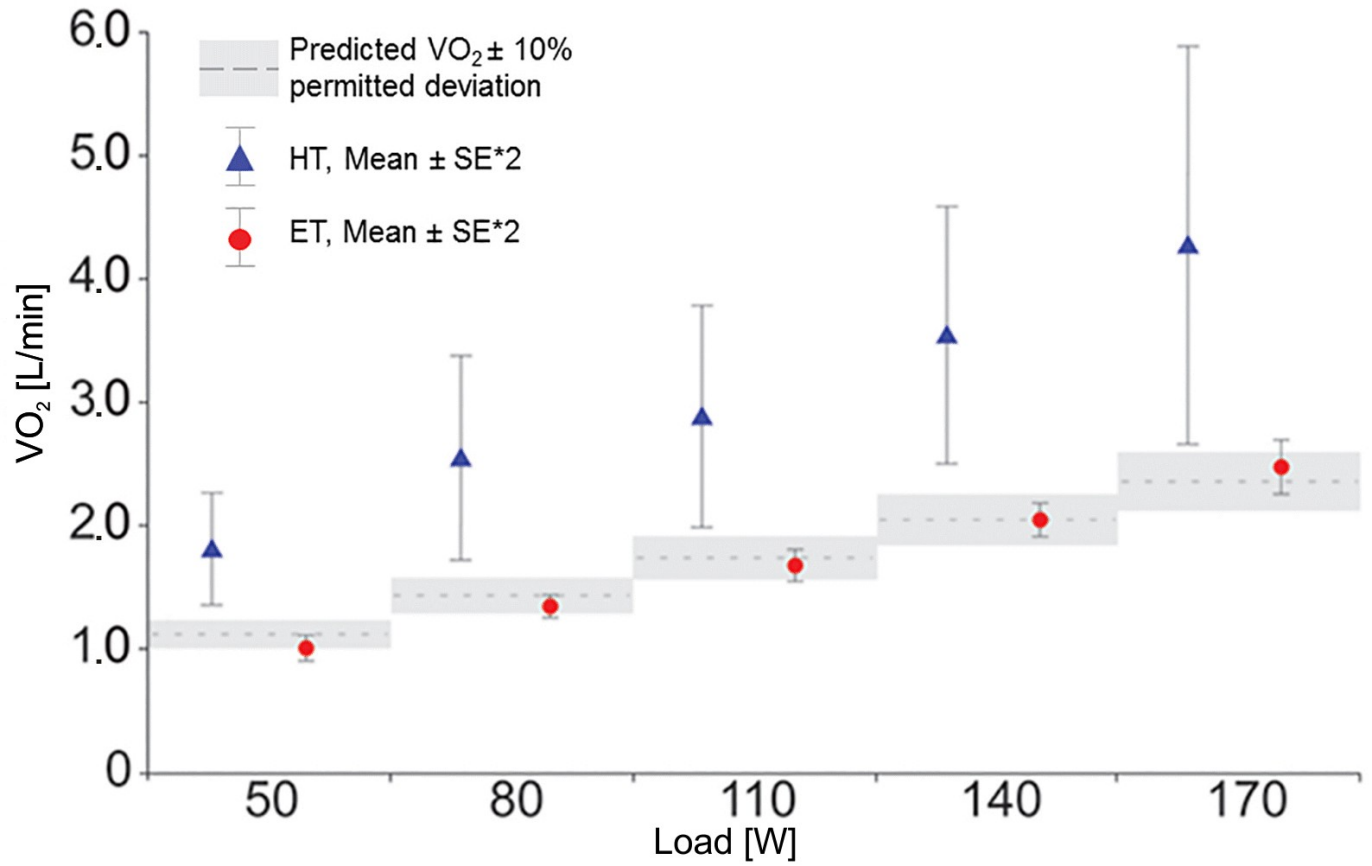
Comparison between HT and ET at FIO_2_ = 78.57%. The dotted lines represent the ${\dot{\text{V}}\text{O}}_{\text{2}}$ predicted for an 80kg man according to Wasserman et al. (2012). The grey block around the dotted line represents the permitted Deviation of ± 10% (see Table 2). The mean values of HT and ET at each load-level are shown with the double standard error to confidence of the plausibility of ${\dot{\text{V}}\text{O}}_{\text{2}}$ predicted. Abbreviations: *HT* Haldane Transformation, *ET* Eschenbacher Transformation.

**Fig 5:**
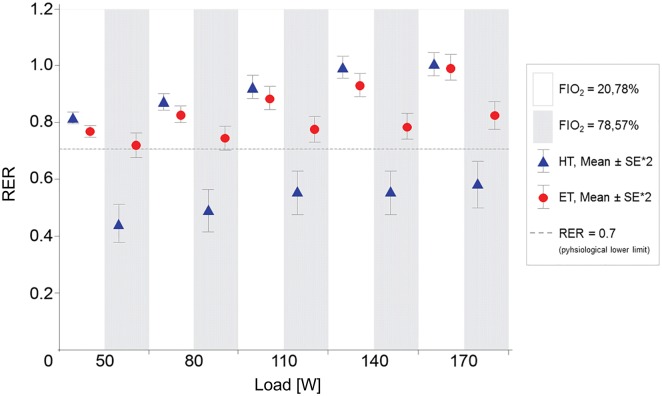
RER comparison at FIO_2_ = 20.78% versus FIO_2_ = 78.57% between HT and ET. HT and ET show plausible RER-values at FIO_2_ = 20.78%. At FIO_2_ = 78.57% there are incomprehensible values of HT (RER < 0.70), while the ET remains constant. RER < 0.70: An RER of 0.70 corresponds to pure fat burning without accompanying carbohydrate metabolism; a lower ratio of carbon dioxide release to oxygen uptake (the RER) is physiologically not possible. Abbreviations: *HT* Haldane Transformation, *ET* Eschenbacher Transformation.

At an FIO_2_ of 78.57%, the HT and ET produced significantly different results (Fig 4). While the RER values calculated with the use of the HT were implausible at < 0.70 over all stress levels, the ET continued to provide physiologically reasonable RER values (Fig 5, Table 3). The results provided by the ET and the HT deviated from the expected ${\dot{\text{V}}\text{O}}_{\text{2}}$ values by 5.1 ± 5.9% and 41.9 ± 3.2%, respectively (Table 3). With the HT, the scattering of individual values increased drastically with increasing load. When the ET was employed, however, a more constant spreading of values over all workload stages was observed (Fig 4), as was also the case at the other tested concentrations of FIO_2_. The t-test showed that, in the case of a calculation by means of the HT, the values were almost significantly different at all loads from the expected values according to Wasserman. Conversely, the values calculated with the ET did not differ significantly from the expected ${\dot{\text{V}}\text{O}}_{\text{2}}$ values (see also Fig 1). The two-sample t-test (HT vs. ET) showed statistically significant differences in the application of the two transformations.

#### Influence of FIO_2_ on the aerobic-anaerobic threshold

Regardless of the transformation method, a shift of the ventilatory threshold in the transition from aerobic to anaerobic cellular respiration was observed with increasing workload in all ten subjects at higher FIO_2_. In tests with an FIO_2_ of 39.85%, the first ventilatory threshold (VT1) did not occur until 140 watts (compared to 125 watts at FIO_2_ = 20.78%). In tests with an FIO_2_ = 78.57%, the first ventilatory threshold (VT1) was reached 136 ± 63 seconds later than that achieved under normal conditions, at a workload of 158 watts. The second ventilatory threshold (VT2) also occurred later (or not at all) in the protocol; in the tests with an FIO_2_ of 39.85%, the VT2 was not observed in seven subjects. The VT2 at an FIO_2_ = 78.57% was not achieved by any of the participants within the test protocol (Table 5). Conversely, under normal conditions (FIO_2_ = 20.78%), the time of VT2 could be determined in six of the ten subjects.

**Table 5:** Mean (SD) of HR, VE and ${\dot{\text{V}}\text{O}}_{\text{2}}\text{max}/\text{kg}$ at 170 watts in ten healthy men. Time shift of reaching the VT1 and VT2 (Mean (SD).

Variables	FIO_2_ = 20.78%		FIO_2_ = 39.85%		FIO_2_ = 78.57%	
HR, 1/min	153 ±19		147 ±20		144 ±19	
VE, L	67 ±15		63 ±13		61 ±17	
${\dot{\text{V}}\text{O}}_{\text{2}}\text{max}/\text{kg}$, (mL/min)/kg	30.9 ±3.2		31.7 ±3.5		34.7 ±7.4	
Variables	VT1 [n = 10]	VT2* [n = 6]	VT1 [n = 10]	VT2* [n = 3]	VT1 [n = 10]	VT2* [n = 0]
t, min:s	05:45 ±00:51	07:57 ±01:31	06:57 ±00:37	08:56 ±00:24	08:01 ±00:52	
Load, W	125 ±15	155 ±15	140 ±0	170 ±0	158 ±15	

* was not achieved by any of the participants within the test protocol. Abbreviations: *HR* heart rate, *VE* ventilation, ${\dot{\text{V}}\text{O}}_{\text{2}}max$ maximum oxygen uptake, *t* time, *VT* ventilatory threshold, *FIO*_*2*_ inspiratory oxygen concentration.

In addition, an adaptation of heart rate (HR), ventilation (VE) and maximum oxygen uptake (${\dot{\text{V}}\text{O}}_{\text{2}}\text{max}$) was observed under higher FIO_2_ conditions, pointing to an improved load adaptation (Table 5).

## Discussion

The spiroergometric examination of persons requiring continuous oxygen supply does not produce meaningful results in terms of ${\dot{\text{V}}\text{O}}_{\text{2}}$ and is therefore rarely applied to this patient group. This is a result of the inaccuracy of the ${\dot{\text{V}}\text{O}}_{\text{2}}$ and RER values from spiroergometric measurements using increased FIO_2_. However, the individual ${\dot{\text{V}}\text{O}}_{\text{2}}$ of a patient can be used to determine his or her performance. Spiroergometry could also contribute to the therapy indication and control, if it provides plausible results that correspond to actual metabolic processes. In the determination of oxygen uptake in particular (and the parameters derived therefrom), this is unfortunately not always possible. The reason for these difficulties lies in the limitation of the Haldane transformation (HT). The calculation of the ${\dot{\text{V}}\text{O}}_{\text{2}}$ using the ‘Eschenbacher transformation’ (ET) is an alternative. In this study, spiroergometry with the ET was investigated under different oxygen concentrations and the hypotheses listed above were tested.

On the basis of the 10 measurements taken under normal conditions (FIO_2_ = 20.78%) and with an FIO_2_ of 39.85%, we could show that both transformations yield plausible ${\dot{\text{V}}\text{O}}_{\text{2}}$ and RER values for these concentrations when compared to values predicted by the Wassermann equation. The mean ${\dot{\text{V}}\text{O}}_{\text{2}}$ values generated with the HT are closer to the predicted values than those determined by the ET (HT = 4.5 ± 1.1%, ET = 8.9 ± 1.6%; HT = 2.2 ± 2.0%, ET = 5.3 ± 4.1%). When the workload increases, however, ${\dot{\text{V}}\text{O}}_{\text{2}}$ values determined with the HT show more scattering than those calculated with the ET. Although not very pronounced at an FIO_2_ = 20.78%, this phenomenon is observed for both FIO_2_ concentrations. Despite this, both transformation equations can be used to generate plausible data for the FIO_2_ concentrations of 20.78% and 39.85%.

The second hypothesis—that the HT does not produce valid ${\dot{\text{V}}\text{O}}_{\text{2}}$ and subsequently RER values at higher concentrations (FIO_2_ > 40%)—can only partially be confirmed and requires further exploration. Investigation into the first hypothesis shows that the use of the HT for ${\dot{\text{V}}\text{O}}_{\text{2}}$ calculation is only acceptable at FIO_2_ values ≤ 40%; oxygen insufflation at higher concentrations result in data inconsistent with predicted values. Indeed, our test results show significant deviation from predicted ${\dot{\text{V}}\text{O}}_{\text{2}}$ values when the HT is employed at FIO_2_ > 80%. Determination of exhaled carbon dioxide (${\dot{\text{V}}\text{CO}}_{\text{2}}$), whose measurement and/or calculation is not influenced by the transformation, however, shows a plausible increase with increasing load. Combined with errors in the calculation of ${\dot{\text{V}}\text{O}}_{\text{2}}$, this leads to an underestimation of RER values, which represent the ratio of ${\dot{\text{V}}\text{CO}}_{\text{2}}$ to ${\dot{\text{V}}\text{O}}_{\text{2}}$. This further confirms that the HT is not suitable for the generation of usable ${\dot{\text{V}}\text{O}}_{\text{2}}$ values during insufflation of oxygen at high concentrations (here FIO_2_ = 80%). The exact concentration of FIO_2_ for which the HT can still validly be employed cannot be definitively deduced from this study. The results obtained show a broad scattering of ${\dot{\text{V}}\text{O}}_{\text{2}}$ data at an FIO_2_ = 40%.

When the ET is applied at increased FIO_2_ concentrations of 40% and 80%, the ${\dot{\text{V}}\text{O}}_{\text{2}}$ measurements correspond to the predicted values, calculated according to Wasserman. Our third hypothesis—that the ET also provides plausible ${\dot{\text{V}}\text{O}}_{\text{2}}$ and RER values at higher levels of FIO_2_ –can thus be confirmed. Furthermore, the scattering of results does not differ significantly for the varying oxygen concentrations. It can therefore be concluded that the ET is a suitable alternative to the HT for measurements at elevated oxygen concentrations.

This is a pilot study with a small number of subjects (n = 10). In future studies this field should be further investigated with patient groups and other forms of increased FIO_2_. Based on the power calculation as suggested by Kuzma (1998) [23], the sample should number at least 27 subjects [24]. Another possible limitation of the study could be the fact that it was performed with a group of healthy men, 36 years of age or younger. The study focuses on the methodical comparison of HT / ET; therefore, it was important to select a homogenous group. Furthermore, it would be useful to study the results with different age and gender groups as well as various diseases.

## Conclusion

As with the classic spiroergometry the spiroergometry under increased oxygen concentration has to generate valid data. However, results of the HT used so far for the calculation of ${\dot{\text{V}}\text{O}}_{\text{2}}$ and RER show an unacceptable variance when using increased FIO_2_ (here 80%). The general application of this method cannot therefore be recommended. The use of the alternative ET at higher FIO_2_ concentrations, however, produces valid results for ${\dot{\text{V}}\text{O}}_{\text{2}}$ and RER calculation. In this study, we could show the accuracy of the ET for FIO_2_ of 40% and 80%. In our opinion, the results of the study presented here allow us to extend the range of applications of spiroergometry. Through the implementation of the ET, spiroergometry at elevated FIO_2_ could provide significant added value for the stress testing of patients with oxygen insufflation.

In measurements with increased FIO_2_, the subjects also showed a shift of the ventilatory thresholds into higher load ranges. The next step, therefore, would be to confirm the findings obtained here in healthy subjects with those of patients suffering from lung disease. The applicability of "spiroergometry under increased oxygen concentration" could be extended and thus also be of benefit to seriously ill patients.

## Supporting information

S1 Appendix Results overview and raw data. (PDF)

S1 Fig Equipment used. This picture is 2018 Vyaire Medical, Inc.; Used with permission (RD_0816-0130).
(TIF)

## Abbreviations

BMI: Body-Mass-Index
ET: Eschenbacher Transformation
FECO_2_: Fraction of Expiratory Carbon dioxide
FEO_2_: Fraction of Expiratory Oxygen
FEV_1_: Forced Expiratory Volume in one second
FICO_2_: Fraction of Inspired Carbon dioxide
FIO_2_: Fraction of Inspired Oxygen
FVC: Forced Vital Capacity
HR: Heart Rate
HT: Haldane Transformation
IC: Inspiratory Capacity
kH: Correction factor of Haldane
RER: Respiratory Exchange Ratio
TLC: Total Lung Capacity
V_E_: Expiratory Volume
V_I_: Inspiratory Volume
${\dot{\text{V}}\text{CO}}_{\text{2}}$: Volume of Carbon dioxide exhalation
VN_2_: Volume of Nitrogen exchange
${\dot{\text{V}}\text{O}}_{\text{2}}$: Volume of Oxygen uptake
