# Supplementary material for: Spiroergometric measurements under increased inspiratory oxygen concentration (FIO2)—Putting the Haldane transformation to the test
Source: PLoS One. 2018 Dec 12;13(12):e0207648. doi: 10.1371/journal.pone.0207648 (PMC6291083; doi:10.1371/journal.pone.0207648)
Supplement: S1 Appendix — (PDF) [file pone.0207648.s001.pdf]

Participants, VO2 and RER

P1 [74Kg]

Eschenbacher Transformation (ET)								
FiO2 %	77,34							
Load-level	averaged VO2 (mL/min)	averaged VCO2 (mL/min)	averaged RER	Plausibility VO2	Deviation VO2 +10%	Deviation VO2 -10%	actual Deviation VO2%	SUM %
50	1340	715	0,53	1095,2	1204,7	985,7	22,4	
80	1479	1007	0,68	1404,2	1544,6	1263,8	5,3	
110	1602	1451	0,91	1713,2	1884,5	1541,9	-6,5	**8,7**
140	2101	1832	0,87	2022,2	2224,4	1820,0	3,9	
170	2636	2286	0,87	2331,2	2564,3	2098,1	13,1	
FiO2 %	39,82							
50	1040	833	0,80	1095,2	1204,7	985,7	-5,0	
80	1204	1059	0,88	1404,2	1544,6	1263,8	-14,3	
110	1646	1613	0,98	1713,2	1884,5	1541,9	-3,9	**5,5**
140	2001	2029	1,01	2022,2	2224,4	1820,0	-1,0	
170	2408	2526	1,05	2331,2	2564,3	2098,1	3,3	
FiO2 %	20,85							
50	975	791	0,81	1095,2	1204,7	985,7	-11,0	
80	1249	1149	0,92	1404,2	1544,6	1263,8	-11,1	
110	1545	1603	1,04	1713,2	1884,5	1541,9	-9,8	**10,0**
140	1832	1977	1,08	2022,2	2224,4	1820,0	-9,4	
170	2130	2310	1,08	2331,2	2564,3	2098,1	-8,6	

Haldane Transformation (HT)								
FiO2 %	77,34							
Load-level	averaged VO2 (mL/min)	averaged VCO2 (mL/min)	averaged RER	Plausibility VO2	Deviation VO2 +10%	Deviation VO2 -10%	actual Deviation VO2%	SUM %
50	3191	715	0,22	1095,2	1204,7	985,7	191,4	**79,4**
80	2665	1007	0,38	1404,2	1544,6	1263,8	89,8	
110	1809	1451	0,80	1713,2	1884,5	1541,9	5,6	
140	3028	1832	0,61	2022,2	2224,4	1820,0	49,7	
170	3747	2286	0,61	2331,2	2564,3	2098,1	60,7	
FiO2 %	39,82							
50	1060	833	0,79	1095,2	1204,7	985,7	-3,2	**4,5**
80	1199	1059	0,88	1404,2	1544,6	1263,8	-14,6	
110	1673	1613	0,96	1713,2	1884,5	1541,9	-2,3	
140	1997	2029	1,02	2022,2	2224,4	1820,0	-1,2	
170	2304	2526	1,10	2331,2	2564,3	2098,1	-1,2	
FiO2 %	20,85							
50	993	791	0,80	1095,2	1204,7	985,7	-9,3	**8,7**
80	1276	1149	0,90	1404,2	1544,6	1263,8	-9,1	
110	1586	1603	1,01	1713,2	1884,5	1541,9	-7,4	
140	1827	1977	1,08	2022,2	2224,4	1820,0	-9,7	
170	2144	2310	1,08	2331,2	2564,3	2098,1	-8,0	

P2 [79Kg]

Eschenbacher Transformation (ET)								
FiO2 %	78,59							
Load-level	averaged VO2 (mL/min)	averaged VCO2 (mL/min)	averaged RER	Plausibility VO2	Deviation VO2 +10%	Deviation VO2 -10%	actual Deviation VO2%	SUM %
50	1107	861	0,78	1124,2	1236,6	1011,8	-1,5	**4,2**
80	1398	1185	0,85	1433,2	1576,5	1289,9	-2,5	
110	1682	1480	0,88	1742,2	1916,4	1568,0	-3,5	
140	1876	1653	0,88	2051,2	2256,3	1846,1	-8,5	
170	2247	2059	0,92	2360,2	2596,2	2124,2	-4,8	
FiO2 %	39,74							
50	1036	773	0,75	1124,2	1236,6	1011,8	-7,8	**5,9**
80	1330	1090	0,82	1433,2	1576,5	1289,9	-7,2	
110	1626	1408	0,87	1742,2	1916,4	1568,0	-6,7	
140	1995	1775	0,89	2051,2	2256,3	1846,1	-2,7	
170	2474	2292	0,93	2360,2	2596,2	2124,2	4,8	
FiO2 %	20,81							
50	1019	785	0,77	1124,2	1236,6	1011,8	-9,4	**7,6**
80	1387	1207	0,87	1433,2	1576,5	1289,9	-3,2	
110	1572	1482	0,94	1742,2	1916,4	1568,0	-9,8	
140	1932	1855	0,96	2051,2	2256,3	1846,1	-5,8	
170	2130	2086	0,98	2360,2	2596,2	2124,2	-9,8	

Haldane Transformation (HT)								
FiO2 %	78,59							
Load-level	averaged VO2 (mL/min)	averaged VCO2 (mL/min)	averaged RER	Plausibility VO2	Deviation VO2 +10%	Deviation VO2 -10%	actual Deviation VO2%	SUM %
50	1762	861	0,49	1124,2	1236,6	1011,8	56,7	**52,8**
80	1978	1185	0,60	1433,2	1576,5	1289,9	38,0	
110	2711	1480	0,55	1742,2	1916,4	1568,0	55,6	
140	3063	1653	0,54	2051,2	2256,3	1846,1	49,3	
170	3875	2059	0,53	2360,2	2596,2	2124,2	64,2	
FiO2 %	39,74							
50	1188	773	0,65	1124,2	1236,6	1011,8	5,7	**5,2**
80	1518	1090	0,72	1433,2	1576,5	1289,9	5,9	
110	1782	1408	0,79	1742,2	1916,4	1568,0	2,3	
140	2125	1775	0,84	2051,2	2256,3	1846,1	3,6	
170	2560	2292	0,90	2360,2	2596,2	2124,2	8,5	
FiO2 %	20,81							
50	1121	785	0,70	1124,2	1236,6	1011,8	-0,3	**3,9**
80	1389	1207	0,87	1433,2	1576,5	1289,9	-3,1	
110	1636	1482	0,91	1742,2	1916,4	1568,0	-6,1	
140	1997	1855	0,93	2051,2	2256,3	1846,1	-2,6	
170	2185	2086	0,95	2360,2	2596,2	2124,2	-7,4	

P3 [83Kg]

Eschenbacher Transformation (ET)								
FiO2 % 78,58								
Load-level	averaged VO2 (mL/min)	averaged VCO2 (mL/min)	averaged RER	Plausibility VO2	Deviation VO2 +10%	Deviation VO2 -10%	actual Deviation VO2%	SUM %
50	1075	672	0,63	1147,4	1262,1	1032,7	-6,3	**8,3**
80	1471	942	0,64	1456,4	1602,0	1310,8	1,0	
110	2031	1246	0,61	1765,4	1941,9	1588,9	15,0	
140	2319	1567	0,68	2074,4	2281,8	1867,0	11,8	
170	2564	2141	0,84	2383,4	2621,7	2145,1	7,6	
FiO2 % 39,60								
50	1096	751	0,69	1147,4	1262,1	1032,7	-4,5	**9,0**
80	1216	1074	0,88	1456,4	1602,0	1310,8	-16,5	
110	1534	1457	0,95	1765,4	1941,9	1588,9	-13,1	
140	1880	1852	0,99	2074,4	2281,8	1867,0	-9,4	
170	2347	2475	1,05	2383,4	2621,7	2145,1	-1,5	
FiO2 % 20,83								
50	936	726	0,78	1147,4	1262,1	1032,7	-18,4	**8,4**
80	1279	1117	0,87	1456,4	1602,0	1310,8	-12,2	
110	1692	1740	1,03	1765,4	1941,9	1588,9	-4,2	
140	1986	2130	1,07	2074,4	2281,8	1867,0	-4,3	
170	2316	2649	1,14	2383,4	2621,7	2145,1	-2,8	

Haldane Transformation (HT)								
FiO2 %	78,58							
Load-level	averaged VO2 (mL/min)	averaged VCO2 (mL/min)	averaged RER	Plausibility VO2	Deviation VO2 +10%	Deviation VO2 -10%	actual Deviation VO2%	SUM %
50	2418	672	0,28	1147,4	1262,1	1032,7	110,7	
80	5179	942	0,18	1456,4	1602,0	1310,8	255,6	
110	4807	1246	0,26	1765,4	1941,9	1588,9	172,3	**150,9**
140	5119	1567	0,31	2074,4	2281,8	1867,0	146,8	
170	4036	2141	0,53	2383,4	2621,7	2145,1	69,3	
FiO2 %	39,60							
50	1243	751	0,60	1147,4	1262,1	1032,7	8,3	
80	1305	1074	0,82	1456,4	1602,0	1310,8	-10,4	
110	1595	1457	0,91	1765,4	1941,9	1588,9	-9,7	**8,4**
140	1898	1852	0,98	2074,4	2281,8	1867,0	-8,5	
170	2262	2475	1,09	2383,4	2621,7	2145,1	-5,1	
FiO2 %	20,83							
50	985	726	0,74	1147,4	1262,1	1032,7	-14,2	
80	1323	1117	0,84	1456,4	1602,0	1310,8	-9,2	
110	1699	1740	1,02	1765,4	1941,9	1588,9	-3,8	**6,9**
140	2009	2130	1,06	2074,4	2281,8	1867,0	-3,2	
170	2276	2649	1,16	2383,4	2621,7	2145,1	-4,5	

P4 [82Kg]

Eschenbacher Transformation (ET)								
FiO2 % 78,47								
Load-level	averaged VO2 (mL/min)	averaged VCO2 (mL/min)	averaged RER	Plausibility VO2	Deviation VO2 +10%	Deviation VO2 -10%	actual Deviation VO2%	SUM %
50	1172	624	0,53	1141,6	1255,8	1027,4	2,7	12,8
80	1587	787	0,50	1450,6	1595,7	1305,5	9,4	
110	1969	1092	0,55	1759,6	1935,6	1583,6	11,9	
140	2243	1177	0,52	2068,6	2275,5	1861,7	8,4	
170	3254	1546	0,48	2377,6	2615,4	2139,8	36,9	
FiO2 % 40,00								
50	1034	806	0,78	1141,6	1255,8	1027,4	-9,4	5,5
80	1418	1203	0,85	1450,6	1595,7	1305,5	-2,2	
110	1778	1518	0,85	1759,6	1935,6	1583,6	1,0	
140	1901	1812	0,95	2068,6	2275,5	1861,7	-8,1	
170	2217	2186	0,99	2377,6	2615,4	2139,8	-6,8	
FiO2 % 20,77								
50	1064	879	0,83	1141,6	1255,8	1027,4	-6,8	9,5
80	1349	1128	0,84	1450,6	1595,7	1305,5	-7,0	
110	1572	1312	0,83	1759,6	1935,6	1583,6	-10,7	
140	1839	1696	0,92	2068,6	2275,5	1861,7	-11,1	
170	2095	2067	0,99	2377,6	2615,4	2139,8	-11,9	

Haldane Transformation (HT)								
FiO2 %	78,47							
Load-level	averaged VO2 (mL/min)	averaged VCO2 (mL/min)	averaged RER	Plausibility VO2	Deviation VO2 +10%	Deviation VO2 -10%	actual Deviation VO2%	SUM %
50	2568	624	0,24	1141,6	1255,8	1027,4	124,9	**205,0**
80	4141	787	0,19	1450,6	1595,7	1305,5	185,5	
110	5491	1092	0,20	1759,6	1935,6	1583,6	212,1	
140	5897	1177	0,20	2068,6	2275,5	1861,7	185,1	
170	9924	1546	0,16	2377,6	2615,4	2139,8	317,4	
FiO2 %	40,00							
50	1176	806	0,69	1141,6	1255,8	1027,4	3,0	**4,5**
80	1595	1203	0,75	1450,6	1595,7	1305,5	10,0	
110	1847	1518	0,82	1759,6	1935,6	1583,6	5,0	
140	2043	1812	0,89	2068,6	2275,5	1861,7	-1,2	
170	2300	2186	0,95	2377,6	2615,4	2139,8	-3,3	
FiO2 %	20,77							
50	1138	879	0,77	1141,6	1255,8	1027,4	-0,3	**6,2**
80	1409	1128	0,80	1450,6	1595,7	1305,5	-2,9	
110	1548	1312	0,85	1759,6	1935,6	1583,6	-12,0	
140	1931	1696	0,88	2068,6	2275,5	1861,7	-6,7	
170	2165	2067	0,95	2377,6	2615,4	2139,8	-8,9	

P5 [90Kg]

Eschenbacher Transformation (ET)								
FiO2 %	79,36							
Load-level	averaged VO2 (mL/min)	averaged VCO2 (mL/min)	averaged RER	Plausibility VO2	Deviation VO2 +10%	Deviation VO2 -10%	actual Deviation VO2%	SUM %
50	825	693	0,84	1188,0	1306,8	1069,2	-30,6	**12,8**
80	1300	1001	0,77	1497,0	1646,7	1347,3	-13,2	
110	1746	1332	0,76	1806,0	1986,6	1625,4	-3,3	
140	2022	1682	0,83	2115,0	2326,5	1903,5	-4,4	
170	2123	2166	1,02	2424,0	2666,4	2181,6	-12,4	
FiO2 %	40,14							
50	943	864	0,92	1188	1306,8	1069,2	-20,6	**13,8**
80	1292	1111	0,86	1497	1646,7	1347,3	-13,7	
110	1598	1531	0,96	1806	1986,6	1625,4	-11,5	
140	1852	1694	0,91	2115	2326,5	1903,5	-12,4	
170	2160	2174	1,01	2424	2666,4	2181,6	-10,9	
FiO2 %	20,70							
50	1160	1007	0,87	1188	1306,8	1069,2	-2,4	**8,9**
80	1360	1088	0,80	1497	1646,7	1347,3	-9,2	
110	1548	1464	0,95	1806	1986,6	1625,4	-14,3	
140	1912	2001	1,05	2115	2326,5	1903,5	-9,6	
170	2207	2471	1,12	2424	2666,4	2181,6	-9,0	

Haldane Transformation (HT)								
FiO2 % 79,36								
Load-level	averaged VO2 (mL/min)	averaged VCO2 (mL/min)	averaged RER	Plausibility VO2	Deviation VO2 +10%	Deviation VO2 -10%	actual Deviation VO2%	SUM %
50	817	693	0,85	1188	1306,8	1069,2	-31,2	**20,6**
80	1088	1001	0,92	1497	1646,7	1347,3	-27,3	
110	1520	1332	0,88	1806	1986,6	1625,4	-15,8	
140	1752	1682	0,96	2115	2326,5	1903,5	-17,2	
170	2150	2166	1,01	2424	2666,4	2181,6	-11,3	
FiO2 % 40,14								
50	980	864	0,88	1188	1306,8	1069,2	-17,5	**13,6**
80	1295	1111	0,86	1497	1646,7	1347,3	-13,5	
110	1577	1531	0,97	1806	1986,6	1625,4	-12,7	
140	1832	1694	0,92	2115	2326,5	1903,5	-13,4	
170	2153	2174	1,01	2424	2666,4	2181,6	-11,2	
FiO2 % 20,70								
50	1233	1007	0,82	1188	1306,8	1069,2	3,8	**5,8**
80	1492	1088	0,73	1497	1646,7	1347,3	-0,3	
110	1677	1464	0,87	1806	1986,6	1625,4	-7,1	
140	1934	2001	1,03	2115	2326,5	1903,5	-8,6	
170	2198	2471	1,12	2424	2666,4	2181,6	-9,3	

P6 [73Kg]

Eschenbacher Transformation (ET)								
FiO2 %	79,02							
Load-level	averaged VO2 (mL/min)	averaged VCO2 (mL/min)	averaged RER	Plausibility VO2	Deviation VO2 +10%	Deviation VO2 -10%	actual Deviation VO2%	SUM %
50	891	685	0,77	1089,4	1198,3	980,5	-18,2	13,4
80	1145	975	0,85	1398,4	1538,2	1258,6	-18,1	
110	1388	1286	0,93	1707,4	1878,1	1536,7	-18,7	
140	1897	1761	0,93	2016,4	2218,0	1814,8	-5,9	
170	2187	2114	0,97	2325,4	2557,9	2092,9	-6,0	
FiO2 %	40,00							
50	1032	800	0,78	1089,4	1198,3	980,5	-5,3	3,6
80	1330	1177	0,88	1398,4	1538,2	1258,6	-4,9	
110	1615	1351	0,84	1707,4	1878,1	1536,7	-5,4	
140	2033	1932	0,95	2016,4	2218,0	1814,8	0,8	
170	2365	2331	0,99	2325,4	2557,9	2092,9	1,7	
FiO2 %	20,68							
50	942	774	0,82	1089,4	1198,3	980,5	-13,5	5,4
80	1386	1141	0,82	1398,4	1538,2	1258,6	-0,9	
110	1613	1397	0,87	1707,4	1878,1	1536,7	-5,5	
140	1920	1780	0,93	2016,4	2218,0	1814,8	-4,8	
170	2267	2211	0,98	2325,4	2557,9	2092,9	-2,5	

Haldane Transformation (HT)								
FiO2 %	79,02							
Load-level	averaged VO2 (mL/min)	averaged VCO2 (mL/min)	averaged RER	Plausibility VO2	Deviation VO2 +10%	Deviation VO2 -10%	actual Deviation VO2%	SUM %
50	1637	685	0,42	1089,4	1198,3	980,5	50,3	21,4
80	1840	975	0,53	1398,4	1538,2	1258,6	31,6	
110	1800	1286	0,71	1707,4	1878,1	1536,7	5,4	
140	2285	1761	0,77	2016,4	2218,0	1814,8	13,3	
170	2480	2114	0,85	2325,4	2557,9	2092,9	6,6	
FiO2 %	40,00							
50	1016	800	0,79	1089,4	1198,3	980,5	-6,7	3,9
80	1360	1177	0,87	1398,4	1538,2	1258,6	-2,7	
110	1608	1351	0,84	1707,4	1878,1	1536,7	-5,8	
140	1962	1932	0,98	2016,4	2218,0	1814,8	-2,7	
170	2363	2331	0,99	2325,4	2557,9	2092,9	1,6	
FiO2 %	20,68							
50	1004	774	0,77	1089,4	1198,3	980,5	-7,8	2,6
80	1418	1141	0,80	1398,4	1538,2	1258,6	1,4	
110	1694	1397	0,82	1707,4	1878,1	1536,7	-0,8	
140	2039	1780	0,87	2016,4	2218,0	1814,8	1,1	
170	2285	2211	0,97	2325,4	2557,9	2092,9	-1,7	

P7 [86Kg]

Eschenbacher Transformation (ET)								
FiO2 %	79,10							
Load-level	averaged VO2 (mL/min)	averaged VCO2 (mL/min)	averaged RER	Plausibility VO2	Deviation VO2 +10%	Deviation VO2 -10%	actual Deviation VO2%	SUM %
50	867	777	0,90	1106,8	1217,5	996,1	-21,7	**11,9**
80	1191	1062	0,89	1415,8	1557,4	1274,2	-15,9	
110	1505	1335	0,89	1724,8	1897,3	1552,3	-12,7	
140	1852	1659	0,90	2033,8	2237,2	1830,4	-8,9	
170	2347	1890	0,81	2342,8	2577,1	2108,5	0,2	
FiO2 %	39,84							
50	937	750	0,80	1106,8	1217,5	996,1	-15,3	**6,7**
80	1366	1137	0,83	1415,8	1557,4	1274,2	-3,5	
110	1677	1428	0,85	1724,8	1897,3	1552,3	-2,8	
140	1982	1743	0,88	2033,8	2237,2	1830,4	-2,5	
170	2563	2238	0,87	2342,8	2577,1	2108,5	9,4	
FiO2 %	20,73							
50	973	818	0,84	1106,8	1217,5	996,1	-12,1	**7,1**
80	1299	1048	0,81	1415,8	1557,4	1274,2	-8,2	
110	1640	1394	0,85	1724,8	1897,3	1552,3	-4,9	
140	1878	1776	0,95	2033,8	2237,2	1830,4	-7,7	
170	2287	2173	0,95	2342,8	2577,1	2108,5	-2,4	

Haldane Transformation (HT)								
FiO2 %	79,10							
Load-level	averaged VO2 (mL/min)	averaged VCO2 (mL/min)	averaged RER	Plausibility VO2	Deviation VO2 +10%	Deviation VO2 -10%	actual Deviation VO2%	SUM %
50	1299	777	0,60	1106,8	1217,5	996,1	17,4	**24,0**
80	1682	1062	0,63	1415,8	1557,4	1274,2	18,8	
110	2182	1335	0,61	1724,8	1897,3	1552,3	26,5	
140	2411	1659	0,69	2033,8	2237,2	1830,4	18,5	
170	3250	1890	0,58	2342,8	2577,1	2108,5	38,7	
FiO2 %	39,84							
50	1054	750	0,71	1106,8	1217,5	996,1	-4,8	**8,9**
80	1548	1137	0,73	1415,8	1557,4	1274,2	9,3	
110	1804	1428	0,79	1724,8	1897,3	1552,3	4,6	
140	2221	1743	0,78	2033,8	2237,2	1830,4	9,2	
170	2732	2238	0,82	2342,8	2577,1	2108,5	16,6	
FiO2 %	20,73							
50	1021	818	0,80	1106,8	1217,5	996,1	-7,8	**4,5**
80	1277	1048	0,82	1415,8	1557,4	1274,2	-9,8	
110	1677	1394	0,83	1724,8	1897,3	1552,3	-2,8	
140	1998	1776	0,89	2033,8	2237,2	1830,4	-1,8	
170	2356	2173	0,92	2342,8	2577,1	2108,5	0,6	

P8 [72Kg]

Eschenbacher Transformation (ET)								
FiO2 %	78,23							
Load-level	averaged VO2 (mL/min)	averaged VCO2 (mL/min)	averaged RER	Plausibility VO2	Deviation VO2 +10%	Deviation VO2 -10%	actual Deviation VO2%	SUM %
50	873	734	0,84	1083,6	1192,0	975,2	-19,4	**9,7**
80	1194	1063	0,89	1392,6	1531,9	1253,3	-14,3	
110	1570	1462	0,93	1701,6	1871,8	1531,4	-7,7	
140	1877	1775	0,95	2010,6	2211,7	1809,5	-6,6	
170	2305	2158	0,94	2319,6	2551,6	2087,6	-0,6	
FiO2 %	39,94							
50	993	788	0,79	1083,6	1192,0	975,2	-8,4	**4,9**
80	1297	1007	0,78	1392,6	1531,9	1253,3	-6,9	
110	1634	1382	0,85	1701,6	1871,8	1531,4	-4,0	
140	1955	1651	0,84	2010,6	2211,7	1809,5	-2,8	
170	2258	2033	0,90	2319,6	2551,6	2087,6	-2,7	
FiO2 %	20,81							
50	979	813	0,83	1083,6	1192,0	975,2	-9,7	**7,2**
80	1241	1181	0,95	1392,6	1531,9	1253,3	-10,9	
110	1632	1475	0,90	1701,6	1871,8	1531,4	-4,1	
140	1903	2005	1,05	2010,6	2211,7	1809,5	-5,4	
170	2460	2353	0,96	2319,6	2551,6	2087,6	6,1	

Haldane Transformation (HT)								
FiO2 %	78,23							
Load-level	averaged VO2 (mL/min)	averaged VCO2 (mL/min)	averaged RER	Plausibility VO2	Deviation VO2 +10%	Deviation VO2 -10%	actual Deviation VO2%	SUM %
50	1215	734	0,60	1083,6	1192,0	975,2	12,1	**11,5**
80	1487	1063	0,71	1392,6	1531,9	1253,3	6,8	
110	1910	1462	0,77	1701,6	1871,8	1531,4	12,2	
140	2297	1775	0,77	2010,6	2211,7	1809,5	14,2	
170	2604	2158	0,83	2319,6	2551,6	2087,6	12,3	
FiO2 %	39,94							
50	1060	788	0,74	1083,6	1192,0	975,2	-2,2	**5,1**
80	1336	1007	0,75	1392,6	1531,9	1253,3	-4,1	
110	1778	1382	0,78	1701,6	1871,8	1531,4	4,5	
140	2176	1651	0,76	2010,6	2211,7	1809,5	8,2	
170	2468	2033	0,82	2319,6	2551,6	2087,6	6,4	
FiO2 %	20,81							
50	1065	813	0,76	1083,6	1192,0	975,2	-1,7	**4,0**
80	1413	1181	0,84	1392,6	1531,9	1253,3	1,5	
110	1707	1475	0,86	1701,6	1871,8	1531,4	0,3	
140	2168	2005	0,92	2010,6	2211,7	1809,5	7,8	
170	2521	2353	0,93	2319,6	2551,6	2087,6	8,7	

P9 [80Kg]

Eschenbacher Transformation (ET)								
FiO2 %	78,28							
Load-level	averaged VO2 (mL/min)	averaged VCO2 (mL/min)	averaged RER	Plausibility VO2	Deviation VO2 +10%	Deviation VO2 -10%	actual Deviation VO2%	SUM %
50	944	515	0,55	1130,0	1243,0	1017,0	-16,5	**10,2**
80	1441	759	0,53	1439,0	1582,9	1295,1	0,1	
110	1817	1058	0,58	1748,0	1922,8	1573,2	3,9	
140	2448	1235	0,50	2057,0	2262,7	1851,3	19,0	
170	2819	1572	0,56	2366,0	2602,6	2129,4	19,1	
FiO2 %	39,58							
50	1101	567	0,51	1130	1243,0	1017,0	-2,6	**9,6**
80	1473	784	0,53	1439	1582,9	1295,1	2,4	
110	2000	988	0,49	1748	1922,8	1573,2	14,4	
140	2356	1352	0,57	2057	2262,7	1851,3	14,5	
170	2701	1685	0,62	2366	2602,6	2129,4	14,2	
FiO2 %	20,79							
50	1070	858	0,80	1130	1243,0	1017,0	-5,3	**8,9**
80	1288	1181	0,92	1439	1582,9	1295,1	-10,5	
110	1531	1394	0,91	1748	1922,8	1573,2	-12,4	
140	1909	1920	1,01	2057	2262,7	1851,3	-7,2	
170	2156	2121	0,98	2366	2602,6	2129,4	-8,9	

Haldane Transformation (HT)								
FiO2 %	78,28							
Load-level	averaged VO2 (mL/min)	averaged VCO2 (mL/min)	averaged RER	Plausibility VO2	Deviation VO2 +10%	Deviation VO2 -10%	actual Deviation VO2%	SUM %
50	1850	515	0,28	1130	1243,0	1017,0	63,7	**160,6**
80	3460	759	0,22	1439	1582,9	1295,1	140,4	
110	4205	1058	0,25	1748	1922,8	1573,2	140,6	
140	6389	1235	0,19	2057	2262,7	1851,3	210,6	
170	8231	1572	0,19	2366	2602,6	2129,4	247,9	
FiO2 %	39,58							
50	1380	567	0,41	1130	1243,0	1017,0	22,1	**36,9**
80	1877	784	0,42	1439	1582,9	1295,1	30,4	
110	2599	988	0,38	1748	1922,8	1573,2	48,7	
140	3004	1352	0,45	2057	2262,7	1851,3	46,0	
170	3249	1685	0,52	2366	2602,6	2129,4	37,3	
FiO2 %	20,79							
50	1131	858	0,76	1130	1243,0	1017,0	0,1	**2,1**
80	1422	1181	0,83	1439	1582,9	1295,1	-1,2	
110	1661	1394	0,84	1748	1922,8	1573,2	-5,0	
140	2088	1920	0,92	2057	2262,7	1851,3	1,5	
170	2298	2121	0,92	2366	2602,6	2129,4	-2,9	

P10 [87Kg]

Eschenbacher Transformation (ET)								
FiO2 % 78,75								
Load-level	averaged VO2 (mL/min)	averaged VCO2 (mL/min)	averaged RER	Plausibility VO2	Deviation VO2 +10%	Deviation VO2 -10%	actual Deviation VO2%	SUM %
50	1069	779	0,73	1170,6	1287,7	1053,5	-8,7	9,3
80	1336	1025	0,77	1479,6	1627,6	1331,6	-9,7	
110	1530	1231	0,80	1788,6	1967,5	1609,7	-14,5	
140	1905	1527	0,80	2097,6	2307,4	1887,8	-9,2	
170	2302	1884	0,82	2406,6	2647,3	2165,9	-4,3	
FiO2 % 39,82								
50	1087	816	0,75	1170,6	1287,7	1053,5	-7,1	8,8
80	1263	885	0,70	1479,6	1627,6	1331,6	-14,6	
110	1659	1283	0,77	1788,6	1967,5	1609,7	-7,2	
140	1918	1490	0,78	2097,6	2307,4	1887,8	-8,6	
170	2255	1776	0,79	2406,6	2647,3	2165,9	-6,3	
FiO2 % 20,79								
50	1123	872	0,78	1170,6	1287,7	1053,5	-4,1	10,7
80	1303	1023	0,79	1479,6	1627,6	1331,6	-11,9	
110	1494	1234	0,83	1788,6	1967,5	1609,7	-16,5	
140	1865	1638	0,88	2097,6	2307,4	1887,8	-11,1	
170	2167	1983	0,92	2406,6	2647,3	2165,9	-10,0	

Haldane Transformation (HT)									
FiO2 %	78,75								
Load-level	averaged VO2 (mL/min)	averaged VCO2 (mL/min)	averaged RER	Plausibility VO2	Deviation VO2 +10%	Deviation VO2 - 10%	actual Deviation VO2%	SUM %
50	1425	779	0,55	1170,6	1287,7	1053,5	21,7	
80	2025	1025	0,51	1479,6	1627,6	1331,6	36,9	
110	2471	1231	0,50	1788,6	1967,5	1609,7	38,2	**39,7**
140	3252	1527	0,47	2097,6	2307,4	1887,8	55,0	
170	3526	1884	0,53	2406,6	2647,3	2165,9	46,5	
FiO2 %	39,82							
50	1148	816	0,71	1170,6	1287,7	1053,5	-1,9	
80	1331	885	0,66	1479,6	1627,6	1331,6	-10,0	
110	1692	1283	0,76	1788,6	1967,5	1609,7	-5,4	**4,2**
140	2087	1490	0,71	2097,6	2307,4	1887,8	-0,5	
170	2327	1776	0,76	2406,6	2647,3	2165,9	-3,3	
FiO2 %	20,79							
50	1164	872	0,75	1170,6	1287,7	1053,5	-0,6	
80	1307	1023	0,78	1479,6	1627,6	1331,6	-11,7	
110	1571	1234	0,79	1788,6	1967,5	1609,7	-12,2	**8,3**
140	1912	1638	0,86	2097,6	2307,4	1887,8	-8,8	
170	2211	1983	0,90	2406,6	2647,3	2165,9	-8,1	

Spirometry Participants

	Age	KG	TLC [L]	TLC [L] (%predicted)	IC [L]	IC [L] (%predicted)	FVC [L]	FVC [L] (%predicted)	FEV 1 [L]	FEV 1 [L] (%predicted)
P1	34	74	6,50	91	3,00	81	5,72	106	4,80	109
P2	29	79	7,54	95	3,78	88	6,79	109	5,36	105
P3	36	83	7,99	106	5,20	132	5,80	102	4,33	95
P4	30	82	7,96	103	3,46	83	5,84	98	4,19	86
P5	27	90	9,49	124	5,88	142	6,27	105	4,43	90
P6	31	73	6,96	94	4,97	127	5,55	98	4,08	88
P7	30	76	9,03	128	4,27	114	6,83	127	5,25	119
P8	29	72	7,50	110	3,27	91	6,18	119	4,95	115
P9	28	80	7,32	105	3,84	103	6,14	115	5,40	122
P10	30	87	7,47	93	4,55	105	5,93	94	4,78	93
	**30**	**80**	**7,78**	**105**	**4,22**	**107**	**6,11**	**107**	**4,76**	**102**
Range -	27	72	6,50		3,00		5,55		4,08	
Range +	36	90	9,49		5,88		6,83		5,40	
SD	3	6	0,90	13	0,92	22	0,43	10	0,49	14

Plausible VO2 according Wasserman [80Kg, man]

Step in W	VO2 plausibel	VO2 +10 %	VO2 -10 %
50	**1128**	1241	1015
80	**1437**	1581	1293
110	**1746**	1921	1571
140	**2055**	2261	1850
170	**2364**	2600	2128

FIO2 – Levels

	FIO_2 - 80%	FIO_2 - 40%	FIO2 - 21%
P1	77,34	39,82	20,85
P2	78,59	39,74	20,81
P3	78,58	39,6	20,83
P4	78,47	40	20,77
P5	79,36	40,14	20,7
P6	79,02	40	20,68
P7	79,10	39,84	20,73
P8	78,23	39,94	20,81
P9	78,28	39,58	20,79
P10	78,75	39,82	20,79
	**78,57**	**39,85**	**20,78**
SD	0,56	0,18	0,06

Summary VO2

VO2 at FIO2 = 20,78%										
	Load-level									
	50		80		110		140		170	
	ET	HT	ET	HT	ET	HT	ET	HT	ET	HT
P1	975	993	1249	1276	1545	1586	1832	1827	2130	2144
P2	1019	1121	1387	1389	1572	1636	1932	1997	2130	2185
P3	936	985	1279	1323	1692	1699	1986	2009	2316	2276
P4	1064	1138	1349	1409	1572	1548	1839	1931	2095	2165
P5	1160	1233	1360	1492	1548	1677	1912	1934	2207	2198
P6	942	1004	1386	1418	1613	1694	1920	2039	2267	2285
P7	973	1021	1299	1277	1640	1677	1878	1998	2287	2356
P8	976	1065	1241	1413	1632	1707	1903	2168	2460	2521
P9	1070	1131	1288	1422	1531	1661	1909	2088	2156	2298
P10	1123	1164	1303	1307	1494	1571	1865	1912	2167	2211
**Mean**	**1024**	**1086**	**1314**	**1373**	**1584**	**1646**	**1898**	**1990**	**2222**	**2264**
SD	77	84	53	72	60	58	46	96	112	112

VO2 at FIO2 = 39,85%										
	Load-level									
	50		80		110		140		170	
	ET	HT	ET	HT	ET	HT	ET	HT	ET	HT
P1	1040	1060	1204	1199	1646	1673	2001	1997	2408	2304
P2	1036	1188	1330	1518	1626	1782	1995	2125	2474	2560
P3	1096	1243	1216	1305	1534	1595	1880	1898	2347	2262
P4	1034	1176	1418	1595	1778	1847	1901	2043	2217	2300
P5	943	980	1292	1295	1598	1577	1852	1832	2160	2153
P6	1032	1016	1330	1360	1615	1608	2033	1962	2365	2363
P7	937	1054	1366	1548	1677	1804	1982	2221	2563	2732
P8	993	1060	1297	1336	1634	1778	1955	2176	2258	2468
P9	1101	1380	1473	1877	2000	2599	2356	3004	2701	3249
P10	1087	1148	1263	1331	1659	1692	1918	2087	2255	2327
**Mean**	**1030**	**1131**	**1319**	**1436**	**1677**	**1796**	**1987**	**2135**	**2375**	**2472**
SD	58	121	84	200	129	298	142	329	168	319

VO2 at FIO2 = 78,57%										
	Load-level									
	50		80		110		140		170	
	ET	HT	ET	HT	ET	HT	ET	HT	ET	HT
P1	1340	3191	1479	2665	1602	1809	2101	3028	2636	3747
P2	1107	1762	1398	1978	1682	2711	1876	3063	2247	3875
P3	1075	2418	1471	5179	2031	4807	2319	5119	2564	4036
P4	1172	2568	1587	4141	1969	5491	2243	5897	3254	9924
P5	825	817	1300	1088	1746	1520	2022	1752	2123	2150
P6	891	1637	1145	1840	1388	1800	1897	2285	2187	2480
P7	867	1299	1191	1682	1505	2182	1852	2411	2347	3250
P8	873	1215	1194	1487	1570	1910	1877	2297	2305	2604
P9	944	1850	1441	3460	1817	4205	2448	6389	2819	8231
P10	1069	1425	1336	2025	1530	2471	1905	3252	2302	3526
**Mean**	**1016**	**1818**	**1354**	**2555**	**1684**	**2891**	**2054**	**3549**	**2478**	**4382**
SD	165	718	146	1309	207	1417	215	1644	348	2584

**SUM VO2**		Load 50W	Load 80W	Load 110W	Load 140W	Load 170W
	Plausible range +10%	1241	1581	1921	2261	2600
	Plausible range -10%	1015	1293	1571	1850	2128
**78,57%**	ET	1016	1354	1684	2054	2478
	HT	1818	2555	2891	3549	4382
**39,85%**	ET	1030	1319	1677	1987	2375
	HT	1131	1436	1796	2135	2472
**20,78%**	ET	1024	1314	1584	1898	2222
	HT	1086	1373	1646	1990	2264

Summary RER

Participant	Transformation	FIO2 (Mean)	RER 50W	RER 80W	RER 110W	RER 140W	RER 170W	Mean RER
P1	ET	78,57%	0,53	0,68	0,91	0,87	0,87	0,77
P2			0,78	0,85	0,88	0,88	0,92	
P3			0,63	0,64	0,61	0,68	0,84	
P4			0,53	0,50	0,55	0,52	0,48	
P5			0,84	0,77	0,76	0,83	1,02	
P6			0,77	0,85	0,93	0,93	0,97	
P7			0,90	0,89	0,89	0,90	0,81	
P8			0,84	0,89	0,93	0,95	0,94	
P9			0,55	0,53	0,58	0,50	0,56	
P10			0,73	0,77	0,80	0,80	0,82	
		**SE*2**	0,14	0,14	0,15	0,16	0,17	
			**0,71**	**0,74**	**0,78**	**0,79**	**0,82**	
P1	HT	78,57%	0,22	0,38	0,80	0,61	0,61	0,53
P2			0,49	0,60	0,55	0,54	0,53	
P3			0,28	0,18	0,26	0,31	0,53	
P4			0,24	0,19	0,20	0,20	0,16	
P5			0,85	0,92	0,88	0,96	1,01	
P6			0,42	0,53	0,71	0,77	0,85	
P7			0,60	0,63	0,61	0,69	0,58	
P8			0,60	0,71	0,77	0,77	0,83	
P9			0,28	0,22	0,25	0,19	0,19	
P10			0,55	0,51	0,50	0,47	0,53	
		**SE*2**	0,20	0,24	0,25	0,26	0,27	
		**Mean**	**0,45**	**0,49**	**0,55**	**0,55**	**0,58**	

P1	ET	39,85%	0,80	0,88	0,98	1,01	1,05	0,84
P2			0,75	0,82	0,87	0,89	0,93	
P3			0,69	0,88	0,95	0,99	1,05	
P4			0,78	0,85	0,85	0,95	0,99	
P5			0,92	0,86	0,96	0,91	1,01	
P6			0,78	0,88	0,84	0,95	0,99	
P7			0,80	0,83	0,85	0,88	0,87	
P8			0,79	0,78	0,85	0,84	0,90	
P9			0,51	0,53	0,49	0,57	0,62	
P10			0,75	0,70	0,77	0,78	0,79	
		**SE*2**	0,10	0,11	0,14	0,13	0,13	
		**Mean**	**0,76**	**0,80**	**0,84**	**0,88**	**0,92**	
P1	HT	39,85%	0,79	0,88	0,96	1,02	1,10	0,79
P2			0,65	0,72	0,79	0,84	0,90	
P3			0,60	0,82	0,91	0,98	1,09	
P4			0,69	0,75	0,82	0,89	0,95	
P5			0,88	0,86	0,97	0,92	1,01	
P6			0,79	0,87	0,84	0,98	0,99	
P7			0,71	0,73	0,79	0,78	0,82	
P8			0,74	0,75	0,78	0,76	0,82	
P9			0,41	0,42	0,38	0,45	0,52	
P10			0,71	0,66	0,76	0,71	0,76	
		**SE*2**	0,13	0,14	0,17	0,17	0,17	
		**Mean**	**0,70**	**0,75**	**0,80**	**0,83**	**0,90**	
P1	ET	20,78%	0,81	0,92	1,04	1,08	1,08	0,92
P2			0,77	0,87	0,94	0,96	0,98	
P3			0,78	0,87	1,03	1,07	1,14	
P4			0,83	0,84	0,83	0,92	0,99	
P5			0,87	0,80	0,95	1,05	1,12	

P6			0,82	0,82	0,87	0,93	0,98	
P7			0,84	0,81	0,85	0,95	0,95	
P8			0,83	0,95	0,90	1,05	0,96	
P9			0,80	0,92	0,91	1,01	0,98	
P10			0,78	0,79	0,83	0,88	0,92	
		**SE*2**	0,03	0,06	0,08	0,07	0,08	
		**Mean**	**0,81**	**0,86**	**0,92**	**0,99**	**1,01**	
P1			0,80	0,90	1,01	1,08	1,08	
P2			0,70	0,87	0,91	0,93	0,95	
P3			0,74	0,84	1,02	1,06	1,16	
P4			0,77	0,80	0,85	0,88	0,95	
P5			0,82	0,73	0,87	1,03	1,12	
P6	HT	**20,78%**	0,77	0,80	0,82	0,87	0,97	**0,88**
P7			0,80	0,82	0,83	0,89	0,92	
P8			0,76	0,84	0,86	0,92	0,93	
P9			0,76	0,83	0,84	0,92	0,92	
P10			0,75	0,78	0,79	0,86	0,90	
		**SE*2**	0,03	0,05	0,08	0,08	0,09	
		**Mean**	**0,77**	**0,82**	**0,88**	**0,94**	**0,99**	

SUM RER	Plausible RER>0,7	Load-level				
FIO2		50	80	110	140	170
78,57%	ET	0,71	0,74	0,78	0,79	0,82
	HT	0,45	0,49	0,55	0,55	0,58
39,85%	ET	0,76	0,8	0,84	0,88	0,92
	HT	0,7	0,75	0,8	0,83	0,9
20,78%	ET	0,81	0,86	0,92	0,99	1,01
	HT	0,77	0,82	0,88	0,94	0,99

Influence of FIO2 on the aerobic-anaerobic threshold

	**VT1**						**Shift of VT1 (t)**		
	**20,78%**		**39,85%**		**78,57%**				
	**t**	**Load**	**t**	**Load**	**t**	**Load**	**20-40**	**40-80**	**20-80**
P1	04:40	110	06:50	140	09:14	170	02:10	02:24	04:34
P2	05:24	110	06:18	140	08:24	170	00:54	02:06	03:00
P3	04:25	110	06:17	140	07:05	140	01:52	00:48	02:40
P4	06:35	140	07:31	140	08:24	170	00:56	00:53	01:49
P5	04:59	110	06:44	140	07:15	140	01:45	00:31	02:16
P6	06:04	140	06:25	140	06:48	140	00:21	00:23	00:44
P7	06:24	140	07:50	140	08:11	170	01:26	00:21	01:47
P8	06:42	140	07:23	140	09:23	170	00:41	02:00	02:41
P9	06:34	140	07:48	140	08:04	170	01:14	00:16	01:30
P10	05:50	110	06:29	140	07:31	140	00:39	01:02	01:41
**Mean**	**05:46**	**125**	**06:57**	**140**	**08:02**	**158**	**01:12**	**01:04**	**02:16**
SD	00:51	15	00:37	0	00:52	15	00:36	00:48	01:03

	VT2						Shift of VT2 (t)		
	20,78%		39,85%		78,57%				
	t	Load	t	Load	t	Load	20-40	40-80	20-80
P1	06:11	140	08:54	170	n.E	n.E	02:43		
P2	09:23	170	n.E	n.E	n.E	n.E			
P3	06:32	140	08:34	170	n.E.	n.E	02:02		
P4	n.E.	n.E.	n.E	n.E	n.E.	n.E.			
P5	07:12	140	09:21	170	n.E.	n.E.	02:09		
P6	09:47	170	n.E.	n.E.	n.E.	n.E.			
P7	n.E.	n.E.	n.E	n.E	n.E.	n.E.			
P8	n.E.	n.E.	n.E	n.E	n.E	n.E			
P9	n.E.	n.E.	n.E	n.E	n.E.	n.E.			
P10	08:36	170	n.E.	n.E.	n.E.	n.E.			
**Mean**	**07:57**	**155**	**08:56**	**170**			**02:18**		
SD	01:31		00:24	0			00:22		

Influence on HR, VE, VO2max

HR, VE, VO2max	KG	FIO2 - 20,78%				FIO2 - 39,85%			
		HR [1/min]	VE [L/min]	VO2max [ml/min]	VO2max/KG [(mL/min)/Kg]	HR [1/min]	VE [L/min]	VO2max [ml/min]	VO2max/KG [(ml/min)/Kg]
P1	74	177	94	2467	33,3	177	88	2592	35,0
P2	79	163	55	2493	31,6	162	52	2613	33,1
P3	83	176	91	2367	28,5	179	78	2417	29,1
P4	82	130	56	2376	29,0	130	53	2411	29,4
P5	90	137	63	2385	26,5	131	65	2373	26,4
P6	73	170	67	2543	34,8	144	64	2388	32,7
P7	76	131	57	2401	31,6	129	56	2614	34,4
P8	72	152	73	2623	36,4	146	69	2443	33,9
P9	80	161	54	2374	29,7	152	47	2902	36,3
P10	87	133	58	2395	27,5	124	54	2330	26,8
**Mean**	**80**	**153**	**67**	**2442**	**30,9**	**147**	**63**	**2508**	**31,7**
SD		19	15	87	3,2	20	13	173	3,5

**HR, VE, VO2max**	**KG**	**FIO2 - 78,57%**			
		HR [1/min]	VE [L/min]	VO2max [ml/min]	VO2max/KG [(ml/min)/Kg]
P1	74	175	77	2712	36,6
P2	79	156	50	2357	29,8
P3	83	174	94	2708	32,6
P4	82	131	44	3713	45,3
P5	90	130	70	2336	26,0
P6	73	140	64	2114	29,0
P7	76	122	49	2674	35,2
P8	72	148	69	2617	36,3
P9	80	142	43	3873	48,4
P10	87	121	51	2381	27,4
**Mean**	**80**	**144**	**61**	**2749**	34,7
SD		19	17	585	7,4
