# Supplementary figures and images for: Spiroergometric measurements under increased inspiratory oxygen concentration (FIO2)—Putting the Haldane transformation to the test
Source: PLoS One. 2018 Dec 12;13(12):e0207648. doi: 10.1371/journal.pone.0207648 (PMC6291083; doi:10.1371/journal.pone.0207648)

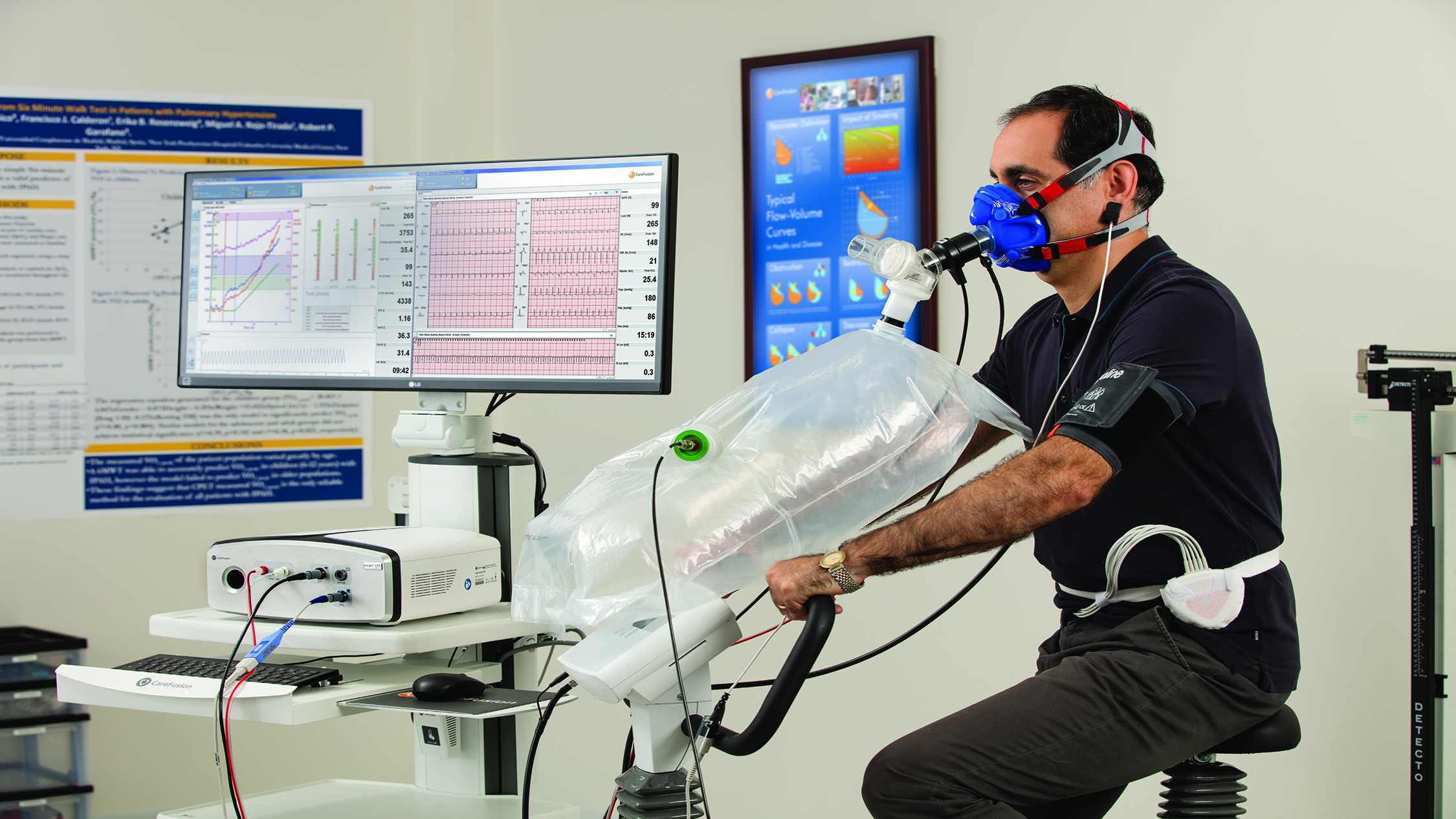

Supplement: S1 Fig — This picture is 2018 Vyaire Medical, Inc.; Used with permission (RD_0816-0130). (TIF) [file pone.0207648.s002.tif]
